# Clinical impact of circulating miR-26a, miR-191, and miR-208b in plasma of patients with acute myocardial infarction

**DOI:** 10.1186/s40001-015-0148-y

**Published:** 2015-06-05

**Authors:** Chencheng Li, Xiaonan Chen, Junwen Huang, Qianqian Sun, Lei Wang

**Affiliations:** Department of Cardiology, the First Affiliated Hospital of Zhengzhou University, Zhengzhou, 450052 China; College of Basic Medical Sciences, Zhengzhou University, Zhengzhou, 450001 China; Department of Clinical Medicine, Zhengzhou University, Zhengzhou, 450052 China; Department of Emergency, the First Affiliated Hospital of Zhengzhou University, Zhengzhou, 450052 China

**Keywords:** Acute myocardial infarction, miR-26a, miR-191, miR-208b

## Abstract

**Background:**

Aberrant expression of several types of miRNAs has been reported in acute myocardial infarction (AMI). The objective of our study was to compare miRNA expression in AMI patients and normal healthy people and determine whether miR-26a, miR-191, and miR-208b could be measured in plasma as indicators for AMI.

**Methods:**

Detection of AMI patients and normal persons by using miRNA microarray chip analysis and miR-26a, miR-191, and miR-208b was screened out. Eighty-seven AMI patients and eighty-seven homogeneous healthy individuals were recruited. Total mRNA including miRNA was isolated and miR-26a, miR-191, and miR-208b expression were determined by qRT-PCR. Receiver operating characteristic curve analysis was performed to evaluate the instructive power of miR-26a, miR-191, and miR-208b for AMI. Dual-luciferase reporter assays indicated p21 is a direct target of miR-208b.

**Results:**

miR-26a and miR-191 were low expressed in AMI compared with normal healthy people, but miR-208b was expressed at a high level in AMI. miR-26a showed an area under the curve (AUC) of 0.745, with a sensitivity of 73.6 % and a specificity of 72.4 %.The AUC for miR-191 was 0.669, with a sensitivity of 62.1 % and a specificity of 69.0 %.The AUC for miR-208b was 0.674, with a sensitivity of 59.8 % and a specificity of 73.6 %.

**Conclusions:**

miR-208b was significantly increased in the AMI compared with healthy people, while miR-26a and miR-191 were decreased. miR-26a, miR-191, and miR-208b were potential indices of AMI, and miR-208b was more effective in patients with non-ST-elevation myocardial infarction.

## Background

Cardiovascular disease has a high mortality rate for men and women worldwide, and acute myocardial infarction (AMI) is the most common cardiovascular disease with high morbidity and mortality [1, 2]. The best known types of acute coronary syndrome are ST-elevation myocardial infarction (STEMI), non-ST-elevation myocardial infarction (NSTEMI), and unstable angina (UA) [3, 4], which cause great harm to human health and financial stress. The door-to-balloon time should not be >90 min, because any delay increases mortality [5]. If a sensitive and specific biomarker could be found to differentiate between AMI and some other causes of chest pain, it could save many lives. Over the past few decades, great progress has been made in the diagnosis and treatment of AMI [6, 7]. However, there is still a need for early diagnosis of AMI in the clinic.

miRNAs are a class of endogenous, single-stranded, 19–22-nucleotide, non-coding RNAs. From the latest database, there are >2000 mature miRNAs associated with the expression of human protein mRNA [8]. miRNAs are involved in multiple biological processes, including proliferation, differentiation, and apoptosis [9–11]. Some researchers have reported that aberrant expression of miRNAs in tissues or cells could promote various diseases, such as cancer and cardiovascular diseases [12–15]. Some studies have indicated that circulating miRNAs in the plasma and serum could act as biomarkers for AMI diagnosis and treatment, such as miR-21, miR-301, miR-328, and miR-34 [16–18].

The purpose of our study was to confirm the level of miR-26a, miR-191, and miR-208b. Some have expressed concern about the relationship between them and AMI [19, 20]. Accordingly, the detection of miRNAs was screened for in AMI patients and the results were compared with a control group that was matched forage, gender, occupation, habits, and ethnicity. We hoped to determine the potential diagnostic value of the three miRNAs; further our understanding of the mechanism of underlying AMI pathogenesis; and contribute to the development of novel, targeted diagnosis and treatment.

## Methods

### Clinical sample collection

This study was approved by the Human Research Ethics Committee of Zhengzhou University, China. We asked every patient to sign the informed consent for the acquisition and use of tissue samples and anonymized the clinical data. A total of 87 AMI patients and 87 normal healthy people were gathered from cases that were treated or examined in the First Affiliated Hospital of Zhengzhou University between 2012 and 2014. There were 32 patients with NSTEMI and 55 with STEMI. Patient characteristics are presented in Table 1. Diagnosis of AMI was based on several indexes: (1) ischemic symptoms; (2) increased levels of troponin and creatine kinase to >2 times the upper limit of normal; (3) ST-segment abnormality; and (4) pathological Q wave [21].The peripheral venous blood samples were immediately frozen after collection and stored in liquid nitrogen until use. Electrocardiography studies were performed by cardiologists. We collected data of disease and individual information through patients and previous material.

**Table 1:** Clinical characters, risk factor, and symptoms of the cohort

Clinicopathological features	AMI cases (*n* = 87)	Normal cases (*n* = 87)	*P*
Age (year)	56.93 ± 9.17	57.28 ± 10.82	>0.05
Male/Female	64/23	62/25	>0.05
Hypertension (Y/N)	38/49	40/47	>0.05
Hypercholesterolemia (Y/N)	9/78	14/73	>0.05
Diabetes (Y/N)	18/69	20/67	>0.05
Alcohol drinking (Y/N)	18/69	17/60	>0.05
Smoking (Y/N)	33/54	39/48	>0.05
Systolic blood pressure (mmHg)	123.88 ± 18.51	130.7 ± 19.0	*0.005*
Diastolic blood pressure (mmHg)	74.68 ± 11.70	80.82 ± 15.8	*0.015*
Heart rate (beats per minute)	74.20 ± 13.03	73.53 ± 13.57	>0.05
Cholesterol (mmol/L)	3.79 ± 0.99	3.79 ± 0.91	>0.05
Triglyceride (mmol/L)	1.39 ± 1.07	1.35 ± 0.92	>0.05
HDL (mmol/L)	1.02 ± 0.21	1.09 ± 0.18	>0.05
LDL (mmol/L)	2.47 ± 0.97	2.51 ± 0.88	>0.05
White blood cells (×10^9^/L)	9.74 ± 3.43	6.93 ± 1.19	<*0.001*
BUN (mmol/L)	6.05 ± 3.55	5.81 ± 1.97	>0.05
CK-MB(U/L)	53.28 ± 8.17	15.61 ± 9.87	<*0.001*
Troponin T (ng/mL)	1.43 ± 1.76	0.05 ± 0.17	<*0.001*

*AMI* acute myocardial infarction, *HDL* high-density lipoprotein, *LDL* low-density lipoprotein, *BUN* blood urea nitrogen, *CK*-*MB* creatine kinase-MB, *P* comparison between AMI patients and healthy controls

Data are expressed as mean ± standard

The results with a significant difference were marked in italics

### Plasma samples

Fasting venous blood samples were collected EDTA tubes. The first blood samples were collected immediately from AMI patients after admission within 4 h of symptom onset, and subsequent blood samples were obtained at admission <4, 24, 48, and 72 h after hospitalization. Samples were centrifuged at 3500× g for 10 min at 4 °C, then the upper solution (plasma) was separated from the cellular layer by pipette and centrifuged at 12,000× g for 10 min at 4 °C, because the 3–5 mm of plasma just above the interphase could help to prevent disturbance of the cellular layer. Plasma was stored at −80 °C until RNA isolation.

### RNA isolation

Total RNA was purified from venous blood samples using the Qiagen RNeasy kit (Valencia, CA, USA).The concentration of RNA was measured using NanoDrop 1000 (Thermo Scientific, Wilmington, DE, USA).

### miRNA microarray chip analysis

Three pairs of AMI plasmas and normal plasmas were detected by microRNA microarray chip analysis. The Agilent miRNA microarray (8 × 60 k) was provided by Shanghai Biotechnology Corporation (Shanghai, China).

### qRT-PCR

qRT-PCR was used to quantify miR-26a, miR-191, and miR-208b expression, according to the Taqman MicroRNA Assays protocol (Applied Biosystems, Foster City, CA, USA). qRT-PCR was performed on an ABI 7500 Fast Real-Time PCR System (Applied Biosystems). U6 was used as an endogenous control. The 2^–ΔCT^ method was used to calculate the relative expression levels of miR-26a, miR-191, and miR-208b in AMI samples compared with non-AMI samples. Experiments were performed in triplicate.

### Dual-luciferase reporter assays

The p21 was amplified from human genomic DNA by PCR, containing putative binding sites for miR-208b, attached to the pmirGLO control vector (Promega, Madison, WI, USA). The 3′UTR of p21 was named pmirGLO-Wt, and pmirGLO-Wt served as the template to occur the pmirGLO-Mut plasmid. Luciferase activity was measured by the dual luciferase assay system (Promega, Madison, WI, USA).

### Statistical analysis

All data required a Kolmogorov–Smirnov normality test, and data are presented as mean ± SD where applicable. Differences between groups were analyzed with the independent two-sample *t* test or Mann–Whitney U test. Receiver operating characteristic (ROC) curve analysis and comparison of the area under the curve (AUC) were performed to estimate the predictive power of biomarkers. All statistical analyses were performed using SPSS version 21.0 (SPSS, Chicago, IL, USA). Differences were considered significant at *P* < 0.05.

## Results

### Characteristics of AMI and normal healthy people

The basic characteristics of the patients and healthy individuals are listed in Table 1. There were no differences between the groups for age, gender, hypertension, hypercholesterolemia, diabetes, alcohol consumption, smoking, heart rate, cholesterol, triglyceride, high-density lipoprotein, low-density lipoprotein, and blood urea nitrogen (*P* > 0.05). Compared with non-AMI cases, AMI cases had significantly lower systolic and diastolic blood pressure (*P* = 0.005 and 0.015). AMI cases had significantly higher white blood cell counts and troponin T level (*P* < 0.001).

### Different expression level of different miRNAs in AMI and normal persons

**Fig. 1:**
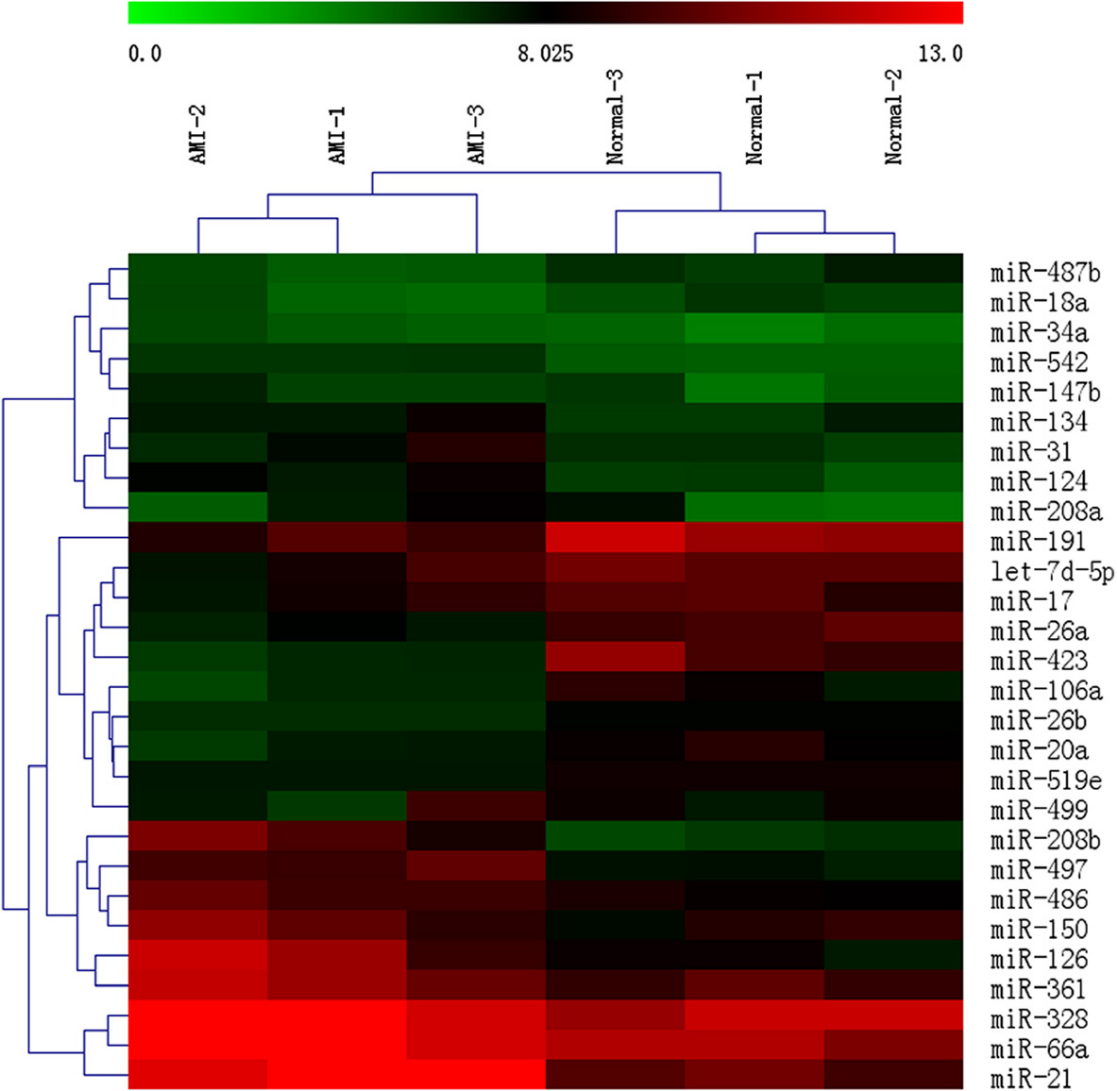
Heat map and hierarchical clustering. The heat map shows the result of the two-way hierarchical clustering of important miRNAs in samples. The *red color* represents a higher relative expression level, and the *green color* represents a lower relative expression level

The results of a two-way hierarchical clustering of the 28 important miRNAs are presented in the heat map (Fig. 1), including 13 miRNAs upregulated and 15 miRNAs downregulated (Table 2). The result indicated that miRNA-208b was significantly increased in AMI cases compared with normal healthy people, while miR-26a and miR-191 were decreased.

**Table 2:** Important miRNAs up- or downregulated in patients with acute myocardial infarction

miRNAs	Regulation	Fold change	miRNAs	Regulation	Fold change
miR-208b	Up	3.58	miR-519e	Down	−2.36
miR-150	Up	3.34	miR-134	Down	−2.41
miR-497	Up	3.27	miR-18a	Down	−2.55
miR-126	Up	3.15	miR-487b	Down	−2.67
miR-66a	Up	3.07	miR-542	Down	−3.03
miR-21	Up	3.00	miR-20a	Down	−3.17
miR-328	Up	2.96	miR-191	Down	−3.21
miR-361	Up	2.89	miR-499	Down	−3.33
miR-147b	Up	2.73	miR-423	Down	−3.45
miR-208a	Up	2.66	miR-106a	Down	−3.5
miR-34a	Up	2.60	miR-26a	Down	−3.88
miR-31	Up	2.52	miR-17	Down	−3.91
miR-124	Up	2.28	let-7d-5p	Down	−3.99
miR-26b	Down	−2.21	miR-486	Down	−4.02

### miR-26a, miR-191, and miR-208b expression in AMI and normal cases within 4 h

**Fig. 2:**
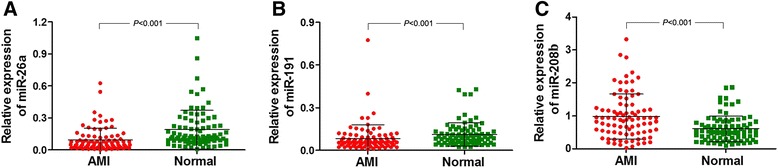
Levels of the three miRNAs in the AMI and normal groups. U6 was used as an endogenous control. **a** miR-26a expression was lower in the AMI group than in the normal group. **b** miR-191 expression was lower in the AMI group than in the normal group. **c** miR-208b was overexpressed in the AMI group compared to the normal group

Expression of the three miRNAs was detected in 87 AMI cases and 87 normal healthy people normalized to U6. The relative expression of miR-26a and miR-191 was significantly decreased in AMI cases compared with normal healthy cases (Fig. 2; *P* < 0.001). In contrast, miR-208b expression was significantly higher in the AMI group (*P* < 0.001).

### Variation in expression of miR-26a, miR-191, and miR-208b with time

**Fig. 3:**
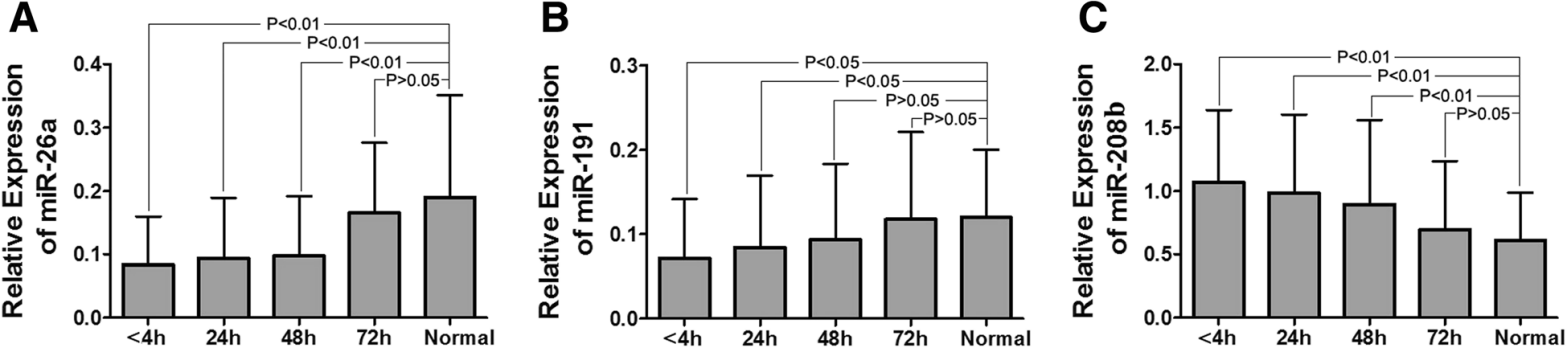
Relative expression of miR-26a, miR-191, and miR-208b over time. Measuring the concentration of miR-26a, miR-191, and miR-208b at several time points. **a** Compared to normal, the AMI patients’ relative expression of miR-26a low expressed 48 h after onset, in 72 h reverted to normal level. **b** Compared to normal, the AMI patients’ relative expression of miR-191 low expressed 24 h after onset, in 48 h reverted to normal level. **c** Compared to normal, the AMI patients’ relative expression of miR-208b overexpressed 48 h after onset, in 72 h reverted to normal level

We showed that the relative expression of miR-26a, miR-191, and miR-208b changed over time. From Fig. 3a, c, we can know that in <4, 24, and 48 h, the differences in expression level of the relative expression of miR-26a and miR-208b between AMI patients and normal were statistically significant (*P* < 0.01); in 72 h, AMI patients reverted to normal level (*P* > 0.05). Figure 3b showed that in <4 and 24 h, the differences of the relative expression of miR-191 between AMI and normal were statistically significant (*P* < 0.01); in 48 h, AMI cases reverted to normal level (*P* > 0.05).

### Diagnostic performance of miR-26a, miR-191, and miR-208b

**Fig. 4:**
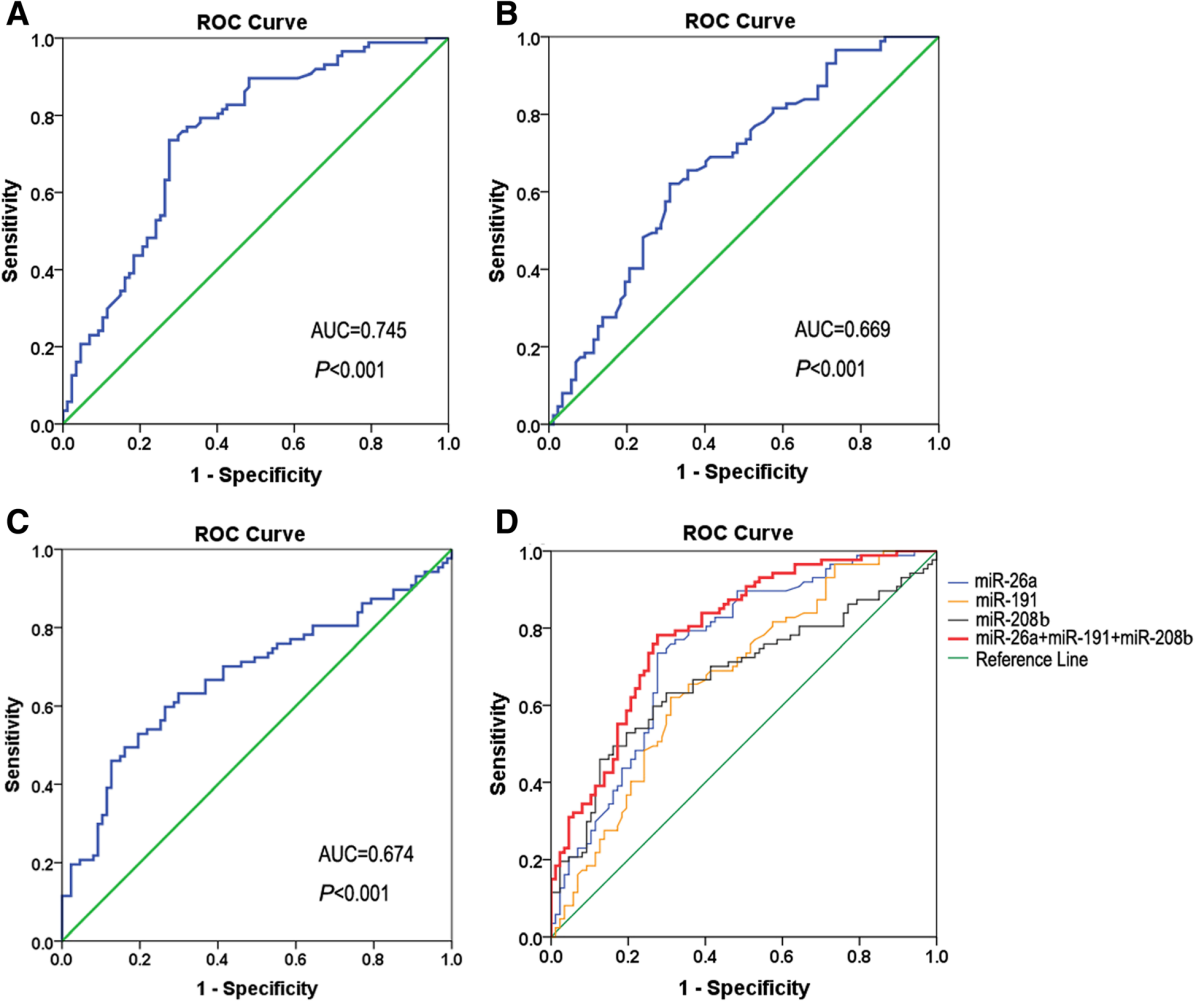
ROC curves for the diagnostic accuracy of the three miRNAs. ROC curve analysis was performed to evaluate the predictive power of miR-26a, miR-191, and miR-208b for AMI. **a** miR-26a with an AUC of 0.745. **b** miR-191 with an AUC of 0.669. **c** miR-208b with an AUC of 0.674. **d** Combination of miR-26a, miR-191, and miR-208b with an AUC of 0.792

ROC curve analysis was performed to evaluate the predictive power of miR-26a, miR-191, and miR-208b for AMI. The ability was determined according to the AUC of 0.745 [95 % confidence interval (CI), 0.671–0.819; *P* < 0.001) for miR-26a (Fig. 4a), 0.669 (95 % CI, 0.589–0.749; *P* < 0.001) for miR-191 (Fig. 4b), and 0.674 (95 % CI, 0.593–0.755; *P* < 0.001) for miR-208b (Fig. 4c). The combination of miR-26a, miR-191, and miR-208 showed an AUC of 0.792 (95 % CI, 0.726–0.858; *P* < 0.001) (Fig. 4d).

### Diagnostic performance of miR-26a, miR-191, and miR-208b for NSTEMI and STEMI

**Fig. 5:**
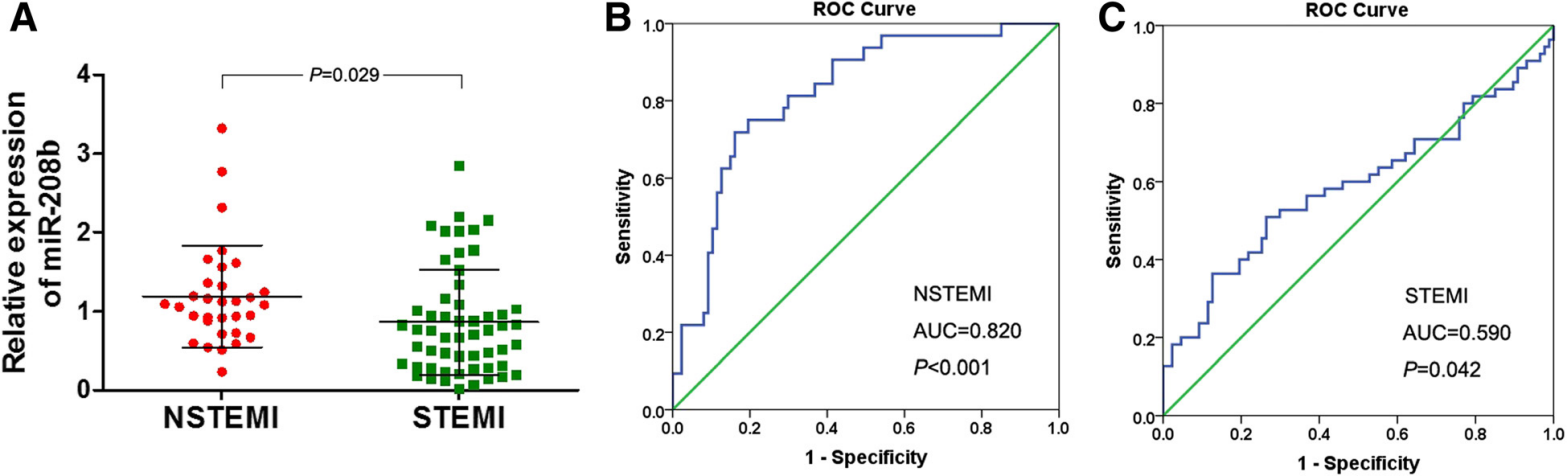
The relationship between miR-208b and NSTEMI and STEMI. **a** Level of miR-208b in NSTEMI and STEMI groups. **b** ROC curve for miR-208b in NSTEMI. **c** ROC curve for miR-208b in STEMI

ROC curve analysis was performed to evaluate the predictive power of miR-208b for NSTEMI and STEMI. The ability was determined according to the AUC of 0.820 (95 % CI, 0.737–0.902; *P* < 0.001) in NSTEMI cases (Fig. 5b) and 0.590 (95 % CI, 0.487–0.692; *P* = 0.042) in STEMI (Fig. 5c). The relative expression of miR-208b was higher in NSTEMI cases compared with STEMI (Fig. 5a). There was no significant difference in the relative expression of miR-26a and miR-191 between the NSTEMI and STEMI groups.

### p21 is a direct target of miR-208b

**Fig. 6:**
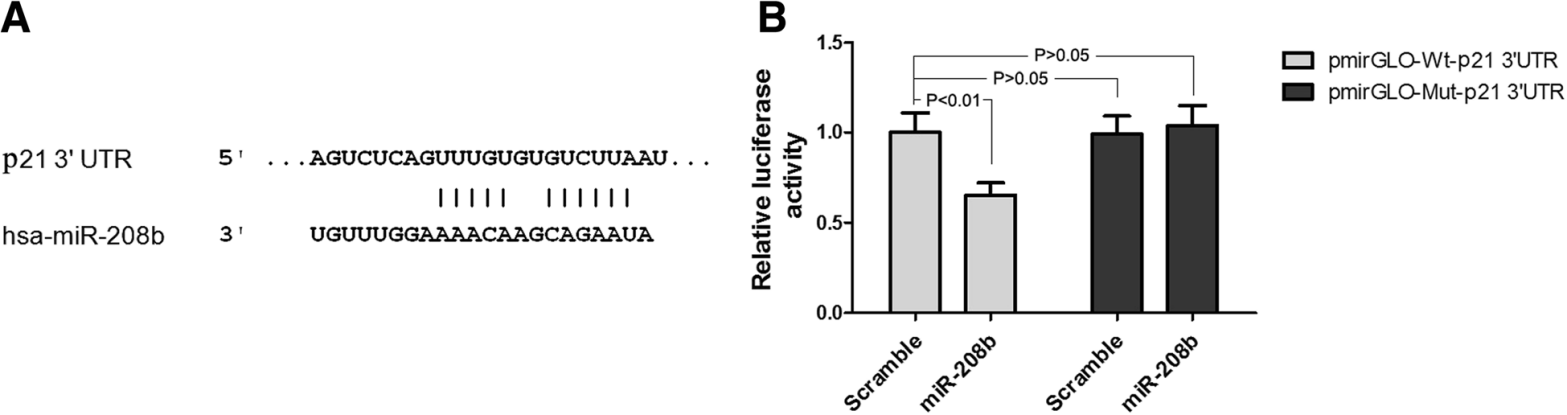
p21 is a target gene of miR-208b. **a** The wild-type p21 3′UTR and p21 3′UTR putative binding sequences in miR-208b. **b** Cotransfection of miR-208b significantly decreased the luciferase activity of the pmirGLO-Wt 3′UTR reporter (*P* < 0.01) but did not decrease that of the pmirGLO-Mut 3′UTR reporter

Using TargetScan and miRanda, analysis indicated that the 3′UTR of p21 contains a predicted seed region for miR-208b (Fig. 6a). To determine whether p21 is regulated by miR-208b, we performed luciferase reporter assays. Cotransfection with miR-208b significantly decreased the luciferase activity of the reporter containing wild-type 3′UTR but did not affect the luciferase reporter with the mutant 3′UTR (Fig. 6b). These data strongly suggest that miR-208b negatively regulates p21 by directly binding to the binding site in the 3′UTR sequence.

## Discussion

There are an increasing number of patients with MI, therefore, early detection and treatment is becoming especially important for AMI. miRNAs are a class of endogenous, small RNA fragments. Although they do not code for proteins, they are involved in many biological processes through signal transduction. They have been used to diagnose cardiac failure and hypertension [22, 23]. Also, some reports have indicated that miRNAs might be effective biomarkers for AMI diagnosis and prognosis [4, 24]. From the results of qRT-PCR, we conclude that miR-208b was significantly overexpressed in AMI patients compared with non-AMI individuals. However, miR-26a and miR-191 were expressed less. ROC curve analysis further showed that miR-26a, miR-191, and miR-208b might be indicators for AMI diagnosis and prognosis.

Recently, circulating miRNAs were found in the plasma [25, 26], and they have high prognostic value after AMI [27, 28]. Human miRNAs separated from plasma are highly conserved at different temperatures but are dissolved in an acid or alkaline environment [29]. A number of studies have shown that the plasma level of miRNAs might affect mortality of AMI patients [30, 31], and the concentration of miRNAs may influence the 1-year survival rate of AMI [32, 33]. In addition, there are several circulating miRNAs that are related to cardiac reconstruction after AMI [34, 35]. These results confirm our view that miRNAs might be indicators for diagnosis and prognosis of AMI, although the mechanisms of diagnostic effect of circulating miRNAs are largely still not clear.

Rapid diagnosis and appropriate treatment of AMI patients are of importance and could have widespread application in clinical practice. So far, cardiac troponins and creatine kinase-MB are the most common biomarkers for AMI diagnosis. However, their availability may be limited in some cases. Thus, the circulating miRNAs might provide a specific biomarker for the diagnosis and treatment of AMI. Our results also showed that the relative expression of miR-208b was higher in NSTEMI compared to STEMI, and more powerful for diagnosis of NSTEMI, according to ROC curves. We suggest that miR-208b is a potential predictor of NSTEMI rather than STEMI.

Previous studies [36] showed that p21 is low expressed in AMI patients within the first 4 h of showing symptoms; from dual-luciferase reporter assays, we got that p21 is a direct target of miR-208b. More important, there is ample evidence that p21 is an important cell cycle inhibitor and can have an effect at the transcriptional level [37]. These results point out that miRNA-208b may function in AMI through p21.

There were some limitations to our study. First, we based our findings mostly on a relatively small sample size, and a larger trial with a more sensitive test is needed to support our results. Second, we only measured the concentrations of miRNAs at one point in time, but continuous measurement made the results more credible. Finally, our method of detection of miRNAs needs to be improved, and to obtain accurate and reproducible results, further research is needed.

## Conclusions

Our data suggested that miR-208b was significantly increased in AMI cases compared with normal healthy people, while miR-26a and miR-191 were decreased. Through ROC curve analyses, we showed that plasma miR-26a, miR-191, and miR-208b were potential indices of AMI, and miR-208b was more effective for NSTEMI.

## Abbreviations

AMI: acute myocardial infarction
EDTA: ethylene diamine tetra acetic acid
ROC: receiver operating characteristic curve
AUC: area under the curve
